# Mammalian Arachidonic Acid 15-Lipoxygenases: Fed-Batch Fermentation, Enzyme Purification and Functional Characterization

**DOI:** 10.3390/metabo16080599

**Published:** 2026-08-21

**Authors:** Vladislav Aksenov, Angelina V. Kurchatova, Alexey Golovanov, Veronika Ulasenko, Olga Zubkova, Alexander Zhuravlev, Ekaterina Makishvili, Nikolay E. Kushlinskii, Hartmut Kuhn, Igor Ivanov

**Affiliations:** 1 Lomonosov Institute of Fine Chemical Technologies, MIREA-Russian Technological University, Vernadskogo pr. 86, 119571 Moscow, Russia; aksenov_v@mirea.ru (V.A.); kurchatova07@mail.ru (A.V.K.); golovanov_a@mirea.ru (A.G.); yakovenko-veronika00@mail.ru (V.U.); olgazubkova0509@mail.ru (O.Z.); alekszhur95@yandex.ru (A.Z.); katamaki@yandex.ru (E.M.); ivanov_i@mirea.ru (I.I.); 2 Blokhin National Research Medical Center of Oncology, Kashirskoe Highway 23, 115478 Moscow, Russia; kne3108@gmail.com; 3 Institute of Biochemistry, Charite-University Medicine Berlin, Corporate Member of Free University Berlin, Humboldt University Berlin and Berlin Institute of Health, Charitéplatz 1, D-10117 Berlin, Germany

**Keywords:** inflammation, mammalian ALOX15, fed-batch fermentation, recombinant proteins, catalytic activity

## Abstract

**Highlights:**

**What are the main findings?**

- Mammalian ALOX isoforms were expressed in mg amounts as catalytically active N-terminal his-tag fusion proteins by fed-batch fermentation in *E. coli*.
- The enzymes were purified to homogeneity and exhibited similar catalytic properties as the wild-type enzymes prepared from natural sources.

**What is the implication of the main finding?**

- The purified proteins are suitable for inhibitor screens, antibody preparation and direct structural analyses.

**Abstract:**

Background: Mammalian arachidonic acid lipoxygenases (ALOXs) are non-heme iron-containing enzymes that oxygenate polyunsaturated fatty acids (PUFAs) with at least two isolated double bonds to hydroperoxy derivatives. The patho-physiological roles of these enzymes in inflammatory, hyperproliferative, and neurological diseases have made them promising targets for pharmacological interventions. Unfortunately, the expression levels of ALOX isoforms in mammalian cells are very low, which makes functional characterization of native enzymes and the development of isoform-specific inhibitors challenging. Methods: Here, we developed a unifying experimental protocol for fed-batch fermentation of mammalian ALOX isoforms in a bioreactor, followed by purification of the recombinant proteins and their functional characterization. Results: Our methodological protocol allowed the preparation of mg amounts of catalytically active human ALOX15, mouse Alox15 and human ALOX15B. The purified proteins are suitable for high-throughput inhibitor screening assays but can also be used as antigens for the preparation of isoform-specific antibodies and for direct structural analyses. Antibodies cross-reacting with human ALOX15 and ALOX15B have been detected in the blood of patients suffering from colorectal cancer. Conclusions: Our functional ALOX data stresses the catalytic differences between mouse and human ALOX15 orthologs, and these catalytic peculiarities need to be considered when the results of mechanistic studies obtained in mouse models of human diseases are transferred to the human situation.

## 1. Introduction

Mammalian ALOXs are non-heme iron-containing enzymes that oxidize polyunsaturated fatty acids (PUFAs), which carry two or more isolated cis double bonds, to the corresponding fatty acid hydroperoxides [1,2]. The human genome contains six functional *ALOX* genes (*ALOX15*, *ALOX15B*, *ALOX12*, *ALOX12B*, *ALOX5*, *ALOXE3*). Human ALOX15 (hALOX15) and human ALOX15B (hALOX15B) oxygenate free arachidonic acid (AA) to the 15-hydroperoxy derivative (15-HpETE). Although the patterns of the AA oxygenation products formed by hALOX15 and hALOX15B are similar, the two enzymes are differentially expressed. The *ALOX15* gene is expressed at high levels in immature red blood cells (reticulocytes), airway epithelial cells, eosinophils [3], and bone-marrow-derived dendritic cells [4]. In contrast, ALOX15B mainly occurs in human skin, the prostate, the lung, and the cornea [5]. Moreover, the protein-chemical and catalytic properties of human ALOX15, on one hand, and human ALOX15B, on the other, are significantly different. Mammalian ALOX15 and ALOX15B orthologs only share a low degree of amino acid identity (some 40%), but their 3D structures are very similar [6,7,8,9,10]. Moreover, hALOX15 exhibits dual reaction specificities with AA as a substrate, and 15(S)-hydroperoxy-5Z,8Z,11Z,13E-eicosatetraenoic acid and 12(S)-hydroperoxy-5Z,8Z,10E,14Z-eicosatetraenoic acid (15(*S*)– and 12(S)–HpETE) are simultaneously formed in a ratio of about 9:1 [11]. In contrast, hALOX15B converts free AA almost exclusively (>99%) to 15(*S*)-HpETE. Mouse Alox15 (mAlox15), which shares a higher degree of amino acid conservation (86%) with hALOX15 [12], functions as an AA 12-lipoxygenating enzyme. It oxygenates free AA predominantly to 12(S)-HpETE (90%), with 15(S)-HpETE being a minor product (10%). Oxygenation of linoleic acid (LA) by human and mouse ALOX15 orthologs and by human ALOX15B leads to the formation of 13(S)-hydroperoxy-9Z,11E-octadecadienoic acid (13(S)–HpODE) [13].

Mammalian ALOX15 orthologs have been implicated in cell differentiation [14,15], ferroptosis [16], insulin resistance, and obesity [17] but their detailed functions are rather complex [18]. In different cancer types and in various inflammation models, ALOX15 and its metabolites exhibit dual functionality. Although ALOX15-derived metabolites may exhibit anti-inflammatory and pro-resolving properties [19,20], the enzymes can also generate pro-inflammatory lipid signals. For instance, the ALOX15/ALOX15B-derived LA metabolite, 13(S)-H(p)ODE, induces suppression of peroxisome proliferator-activated receptor γ (PPARγ) expression and stimulates tumor growth in prostate cancer [21]. The biological function of ALOX15B also remains controversial. The enzyme exhibits tumor-suppressive activities in prostate and breast carcinomas [22], but in colorectal cancer, expression of this enzyme has been associated with poorer prognosis [23].

Different ALOX isoforms suitable for high-throughput inhibitor screenings have previously been prepared in *E. coli* [24] and *Sf9* insect cells [25,26]. The major limitations of these systems are the high costs. Moreover, several ALOX isoforms expressed in *Sf9* insect cells lose their catalytic activities upon freezing and thawing. Although *E. coli* strains represent a classic platform for recombinant protein expression, the production of sufficient quantities of mammalian ALOX isoenzymes is frequently limited by the formation of inclusion bodies. Potential ways to solve these problems include (i) scaling up fed-batch cultivation in a bacterial fermenter; (ii) optimization of protein induction without modification of the target plasmid sequence; and (iii) renaturation of misfolded proteins.

In this paper, we describe a unifying protocol for fed-batch fermentation of mammalian ALOX isoforms (hALOX15, hALOX15B, mAlox15) in a 7 L fermenter. The recombinant proteins were purified to apparent electrophoretic homogeneity from the bacterial lysis supernatant using two types of consecutive chromatography (affinity chromatography on a Ni resin and IEX Q20 IEX Q20 anion exchange chromatography). The purified enzymes were characterized with respect to their enzymatic properties and their sensitivity against octyl (N-(5-(1H-indol-2-yl)-2-methoxyphenyl)sulfamoyl)carbamate, a substrate-selective inhibitor of rabbit ALOX15 [27].

## 2. Materials and Methods

### 2.1. Reagents

The chemicals used in this study were from the following sources: Tris(hydroxymethyl)aminomethane base, acetic acid (reagent grade), bromophenol blue, inorganic salts (extra pure grade), methanol and ethanol from Khimprom-M (Moscow, Russia); imidazole, ethylenediaminetetraacetic acid (EDTA), acrylamide, N’,N’-bismethylene acrylamide and tetramethylethylenediamine (TEMED) from Dia-M (Moscow, Russia); Tween-20 and β-mercaptoethanol (β-ME) from Neofroxx (Darmstadt, Germany); sodium dodecyl sulfate (SDS) and ammonium persulfate (APS) from Sigma (St. Louis, MO, USA); glycerol (special purity grade) from Helicon (Moscow, Russia); complex peptone medium from Greenvan (Moscow, Russia); glucose from BioMir (Moscow, Russia); lactose from Armor Proteines (Saint-Brice-en-Coglès, France); antifoam reagent from Sofeks-silikon (Moscow, Russia); and isopropyl β-D-1-thiogalactopyranoside (IPTG) and kanamycin sulfate salt from Panreac AppliChem (Barcelona, Spain). Affinity sorbent Ni-TED was purchased from Sunresine (Xian Shaanxi, China), Ni-NTA agarose from Serva (Heidelberg, Germany) and Ni-IMAC from BioToolomics (Consett, UK). Coomassie G-250 and BlueBlock were obtained from Serva (Heidelberg, Germany), HRP-conjugated rabbit anti-His-Tag mAb (AE104) from Abclonal (Düsseldorf, Germany) and HRP-conjugated mouse antibodies (P-MGH Igg) from IMTEK (Moscow, Russia). HRP substrate (G2014-100ML) was purchased from Servicebio (Wuhan, Hubei, China). For SDS-PAGE, a pre-colored mixture of molecular weight markers (10–200 kDa) was purchased from Servicebio (Wuhan, Hubei, China). An unstained marker mixture (12–80 kDa) was obtained from TransGen Biotech (Beijing, China).

### 2.2. Vector Construction and Preparing Working Cell Banks

The coding regions of hALOX15 and mAlox15 were cloned into the *pET28b(+)* expression vector between the *SalI* (N-terminus) and *HindIII* (C-terminus) restriction sites as described previously [24]. For expression of hALOX15B, the *pET28b(+)* expression vector was also used, but here, the coding region was inserted between the *BamHI* and *HindIII* restriction sites. *E. coli BL21 (DE3) pLysS* chemically competent cells (Evrogen, Moscow, Russia) were transformed with the *pET28b(+)-hALOX15*, the *pET28b(+)-hALOX15B*, and the *pET28b(+)-mAlox15* constructs. For bacterial expression, the competent zs were prepared, frozen, and stored in the presence of 15% (*v*/*v*) glycerol at −80 °C.

### 2.3. Bacterial Cultivation in Liquid Cultures

The liquid cultures were inoculated with 0.1% (*v*/*v*) glycerol cell stocks in 25 mL of LB broth medium (100 mL flasks), with the addition of 50 µg/mL kanamycin sulfate and 3.0 g/L of glucose, and were incubated overnight at 200 rpm and 37 °C. The main cultures were inoculated with 1% (*v*/*v*) of the overnight cultures in 500 mL (1 L flasks) with the addition of the appropriate antibiotics and 3 g/L glucose. Cell cultures were grown up to optical density at 600 nm (OD_600_) = 0.8 at 200 rpm and 37 °C. Induction of ALOX isoform expression was conducted either by 2.7% (*w*/*v*) lactose, 1 mM IPTG, or by a mixture of alternative inducers [0.25 mM IPTG and 2.7% (*w*/*v*) lactose)] for 6 h at 25 °C.

### 2.4. Bacterial Cultivation in a Fermenter

The inoculation medium was a peptone solution (15.0 g/L) containing 10.0 g/L NaCl, 5.0 g/L and 0.1 g/L kanamycin sulfate. The medium was supplemented with 0.1% (*v*/*v*) working bank cells and incubated at 37 °C, 200 rpm, overnight. The fermenter medium was composed of 42.7 g/L complex peptone medium, 5.0 g/L NaCl, 3.0 g/L glucose, 0.5 g/L MgSO_4_·7H_2_O, 6 g/L K_2_HPO_4_·3H_2_O and 1.3 g/L Sofexil anti-foam reagent. The lactose feed solution contained lactose at a concentration of 190.0 g/L, 154.4 g/L glycerol, 5.8 g/L MgSO_4_·7H_2_O and 5.0 g/L NH_4_H_2_PO_4_. The working volume of the lactose solution was 500 mL. The antifoam solution contained 50 g/L antifoam reagent. The trace element solution consisted of the following salts and chemicals: ZnCl_2_ (9.9 mg/L), FeCl_3_·6H_2_O (44.1 mg/L), CoCl_2_·6H_2_O (4.0 mg/L), Na_2_MoO_4_·2H_2_O (4.0 mg/L), MnCl_2_·4H_2_O (23.5 mg/L), CuSO_4_ (2.2 mg/L), H_3_BO_3_ (4.7 mg/L), and EDTA (813 mg/L) [28]. The pH of the trace element solution was adjusted to pH 11 using a 25% (*v*/*v*) ammonia solution. This trace element solution was added to the lactose solution. Culture media, lactose solution and the salt solutions were separately sterilized by autoclaving at 121 °C for 30 min. Immediately before the fermentation process, sterile-filtered kanamycin sulfate solution (100 mg/L) was added.

The fermentation process was carried out in a 7 L glass laboratory fermenter (Novaferm AB, Falkenberg, Sweden) with a working volume of 3.5 L at 37 °C, 1 VVM air flow, pH 7.0, and 450 rpm of initial stirring speed. The oxygen concentrations (pO_2_) were manually kept constant between 35 and 40% saturation by modulation of the stirring speed. The inoculum volume was 3% (*v*/*v*). The pH value was maintained using 30% orthophosphoric acid or 25% ammonia solutions. Foaming was controlled using a foam sensor and automatic feeding of supplementary “Sofexil” antifoam solution. Induction of ALOX isoform biosynthesis was conducted either by 1 mM IPTG or by a mixture of different inducers (0.25 mM IPTG and 2.7% (*w*/*v*) lactose) for 6 h at 25 °C. At the end of the fermentation process, bacteria were pelleted using the Bioridge H2500R centrifuge (Bioridge, Shanghai, China) at 10,000× *g* (10 min, 4 °C), and the resulting biomass was stored at −75 °C.

Bacterial biomass homogenization was performed using a high-pressure homogenizer Nanogenizer (Genizer LLC, Irvine, CA, USA) at 800 bars for 3 cycles at 4 °C. The presence of intact cells was quantified by light microscopy. All buffers were filtered sterile via a 0.22 µm membrane. The biomass was suspended in a buffer for homogenization (50 mM PBS, 0.1% Tween-20, pH 7.2) at a ratio of 1:5. The cell lysate was centrifuged at 10,000× *g* for 10 min at 4 °C. The cell-free homogenate supernatants were used as a source for enzyme purification.

### 2.5. Metal-Affinity Chromatography and Anion Exchange Chromatography

The clear lysate supernatant from 20 g of biomass was subjected to affinity chromatography (bed volume 1 mL). The column was washed (2 × 10 mL) with buffer A (50 mM KH_2_PO_4_, 300 mM NaCl, pH 8.0), 4 mL of buffer B (50 mM KH_2_PO_4_, 300 mM NaCl, 10 mM imidazole, pH 8.0) and 4 mL of buffer C (50 mM KH_2_PO_4_, 300 mM NaCl, 25 mM imidazole, pH 8.0). The bound His-tag fusion proteins were eluted with buffer D (50 mM KH_2_PO_4_, 300 mM NaCl, 200 mM imidazole, pH 8.0). Five 300 μL fractions were collected, and the ALOX activity was assayed. The fractions containing catalytically active ALOX protein were pooled and desalted using 5 mL HiTrap™ desalting columns (Cytiva, Uppsala, Sweden). For final purification of the recombinant proteins, we used an ÄKTA FPLC system (Cytiva, Uppsala, Sweden). Anion exchange chromatography was performed on a 1 mL BioPro SmartSep IEX Q20 column (YMC, Kyoto, Japan). The desalted protein solutions (up to 5 mL) were loaded into the column and eluted with a linear gradient of buffers A and B. Buffer A: 20 mM Tris–HCl (pH 8.0), buffer B: 20 mM Tris, 1 M NaCl (pH 8.0). Fractions of 1 mL were collected, and those containing active LOX protein were pooled.

### 2.6. Immunoblotting

Following SDS-PAGE in 10% separating gels, the gel and a nitrocellulose membrane were incubated in electrode buffer with 30% MeOH for 5 min. A sandwich was assembled to transfer proteins from the gel to the membrane, and the transfer was performed by the EZ Blot Semi-Dry Transfer System (e-BLOT Life Science Co., Ltd., Shanghai, China) at 1 A for 12 min. Finally, the membrane with the transferred proteins was blocked using a 1× BlueBlock aqueous solution at 25 °C for 1 h and incubated with a 1:5000 diluted (*v*/*v* according to manufacturer recommendation) HRP-conjugated rabbit anti-His-Tag antibody in 1× BlueBlock aqueous solution at 25 °C for 1 h. The membrane was washed three times with 1× TBST buffer (10 mM Tris-HCl, 150 mM NaCl, 0.05% Tween-20, pH 7.2) for 10 min, then with water, and incubated with HRP substrate (1:1, *v*/*v* according to manufacturer recommendations) for 2 min. Chemiluminescence signals were registered with the ChemiScope 6200 Touch chemodocumentary system (Clinx, Shanghai, China), with a 5 to 300 ms exposure time.

### 2.7. ALOX15 Activity Assay

For quantification of the reaction kinetics, the oxygenation of AA and LA by the different ALOX isoforms was assayed spectrophotometrically using a SPECORD 50 Plus spectrophotometer (Analytik Jena GmbH Co KG, Jena, Germany), measuring the increase in absorbance at 235 nm in the substrate concentration range of 1–50 μM. Both AA and LA were prepared as Na^+-^salt stock solutions in PBS (5 mM). The assay mixture was in PBS (pH 7.2) that contained different ALOX concentrations (2–5 µg/mL, final concentration) depending on the specific activity of the enzyme preparations. The reaction was started by the addition of substrate. All measurements were carried out at room temperature.

The impact of the inhibitor on the rate of LA or AA oxygenation was also assayed spectrophotometrically by measuring the increase in conjugated diene formation (235 nm). The assay mixture was a 50 mM phosphate buffer (pH 7.2) which contained the inhibitors at different concentrations. No detergents were used in any of our kinetic assays. Octyl (N-(5-(1H-indol-2-yl)-2-methoxyphenyl)sulfamoyl)carbamate was dissolved in DMSO, and serial dilutions of the stock solutions with DMSO were carried out so that from each dilution, 1 μL was applied to the assay sample. Purified enzymes (2–5 μg) were pre-incubated with the inhibitor for 1 min, and the reaction was started by the addition of substrate. The linear part of the kinetic progress curve was evaluated. The activity of the solvent controls (1 µL DMSO) was set as 100%.

### 2.8. HPLC-MS Analysis

Analysis of the PUFA oxygenation products was performed using an Agilent 1260 Infinity II LC-MS system that was equipped with a quaternary gradient pump, an autosampler, a column oven, a variable-wavelength UV detector, and a single quadrupole mass-selective detector LC/MSD iQ. Separation of the analytes was carried out at 30 °C on a Nucleodur C18 gravity column (Macherey-Nagel, Düren, Germany; 250 × 4 mm, 3 μm particle size) coupled with a guard column (6 × 4 mm, 3 μm particle size). The mobile phase consisted of water containing 0.1% formic acid (phase A) and acetonitrile containing 0.1% formic acid (phase B). Elution was performed with a linear gradient of phase B at a flow rate of 1.0 mL/min according to the following program: 0 min—10% B; 2 min—10% B; 15 min—100% B; 20 min—100% B. The injection volume was 25 µL. The flow was split in a 4:1 ratio post-column, with one portion (0.2 mL/min) directed into the mass-sensitive detector.

Mass spectrometric detection was performed using electrospray ionization (ESI) combined with selective ion monitoring (SIM). The ion source parameters were as follows: gas temperature of 325 °C, gas flow rate of 8 L/min, nebulizer pressure of 40 psi, and capillary voltage of 4000 V. For each ion, a dwell time of 333 ms and a fragmentor voltage of 150 V were applied. The monitored ions were m/z 295 (13-HODE) and m/z 319 (12- and 15-HETE).

### 2.9. Enzyme-Linked Immunosorbent Assay (ELISA)

The recombinant ALOX isoforms (100 µL of 10 µg/mL solution per well) were immobilized in the wells of a 96-well plate (ThermoFisher, Waltham, MA, USA) at 4 °C overnight. Then the wells were blocked with a 1% BSA solution (Dia-m, Moscow, Russia) for 1 h at 25 °C. The blood sera of patients suffering from colorectal cancer (stages I and II) were obtained from the Blokhin National Research Medical Center of Oncology (Moscow, Russia). Control sera of healthy volunteers were also obtained from this institution. The sera were added to the blocking buffer at a dilution of 1:100. HRP-conjugated mouse antibodies in working dilutions of 1:5000 to total human antibodies and tetramethylbenzidine as a substrate were used. The optical density was determined using a Siemens BEP2000 Advance analytical system (Deerfield, IL, USA).

### 2.10. Miscellaneous

ALOX protein concentration was determined using the Bradford protein assay, employing purified rabbit ALOX15 as a standard (specific extinction coefficient of 1.78 mL mg^−1^ cm^−1^) [11]. GraphPad Prism 8 and the Michaelis–Menten equation were used for calculation of the kinetic parameters.

## 3. Results

### 3.1. Bacterial Expression of Mammalian ALOX Isoforms

**Figure 1:**
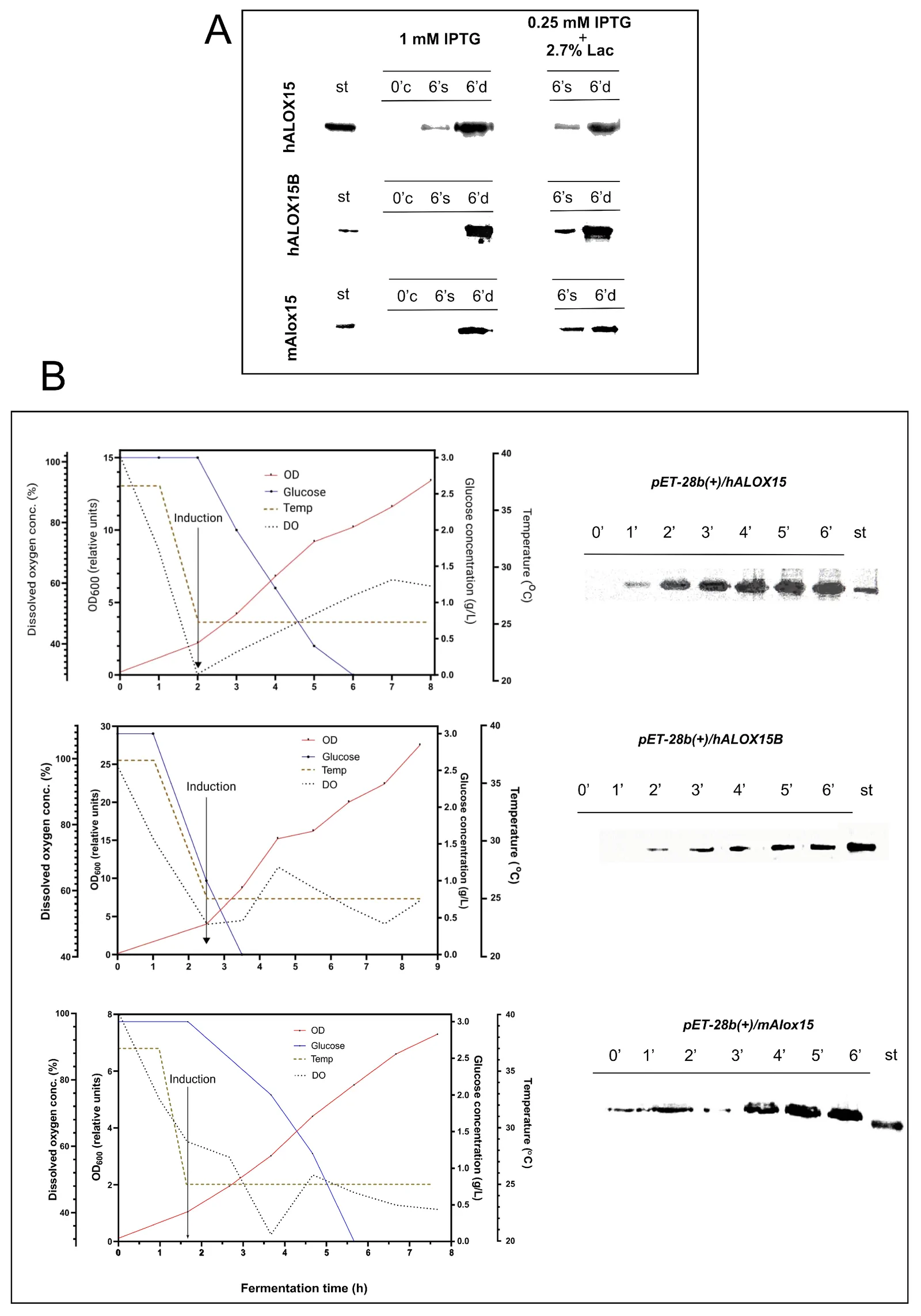
Expression of recombinant ALOX isoforms. (**A**) Bacterial expression of ALOX isoforms in the *E. coli BL21(DE3) pLysS* producer cells. (**B**) Bacterial expression of hALOX15 (upper panel), hALOX15B (middle panel), and mAlox15 (lower panel) in *E. coli BL21 (DE3) pLysS* with IPTG + lactose as inducer in a 7 L laboratory fermenter. Protein expression and solubility analysis were performed by immunoblotting with horseradish peroxidase-conjugated rabbit monoclonal antibodies against the His-tag. All details are described in Materials and Methods section. Legend: st—pure recombinant hALOX15 used as a standard, 0′–6′—induction time in hours, c—total cell suspension, s—bacterial lysate supernatant, d—debris (bacterial lysate pellet).

At the initial stage of this study, we optimized both the growth and protein expression conditions for the recombinant *E. coli BL21 (DE3) pLysS/pET28b(+)-hALOX15/KANr* and the *E. coli BL21 (DE3) pLysS/pET28b(+)-hALOX15B/KANr* producer strains in liquid culture flasks. Heterologous recombinant proteins are predominantly expressed in the early log phase of the growth kinetics [29], and we selected an optical density OD_600_ of 0.8 to test the expression of the recombinant ALOX isoforms. Temperature is an important parameter for both microbial growth and protein biosynthesis. The optimal growth temperature for most *E. coli* strains is 37 °C. However, at this temperature, protein expression is frequently compromised; thus, we selected 25 °C as the expression temperature for all experiments. Lower expression temperatures (20 °C) were sub-optimal, as biomass formation was reduced. To maximize the expression of recombinant ALOX isoforms (Figure 1A), we tested three different expression inducers: (i) 1 mM IPTG, (ii) 2.7% (by weight) lactose, and (iii) a mixture of 0.25 mM IPTG and 2.7% (by weight) lactose. Unfortunately, we did not observe any expression of hALOX15 or hALOX15B when lactose was used as the sole inducer. This finding may be related to the relatively weak ability of lactose to inactivate the LacI repressor and to the high consumption of this substrate as a carbon and energy source. In contrast, in the presence of 1 mM IPTG and when the IPTG–lactose mixture was used as an expression inducer, large amounts of recombinant proteins were detected after a 6 h incubation period (Figure 1A, upper panel).

hALOX15B and mAlox15 were also expressed when the IPTG–lactose mixture was used as an expression inducer (Figure 1A, lower panels). The maximum accumulation of both proteins in soluble form was achieved after a 6 h induction period at 25 °C. Although lactose appears to be a less stable inducer owing to its consumption as an energy source, it extends the exponential growth phase, making the system more suitable for fed-batch fermentations. Based on this expression data, we next scaled up the expression system for a 7 L fermenter using the IPTG–lactose mixture as an expression inducer (Figure 1B).

Since the soluble form of hALOX15 was formed either with pure IPTG or with the IPTG–lactose mixture, both conditions were compared for protein biosynthesis (Supporting Information, Figure S1A). Here, we found that application of the IPTG–lactose mixture resulted in a 14% higher biomass formation. Moreover, the relative enzymatic activity of recombinant hALOX15 and the amounts of ALOX15 protein were 50% higher than those for the enzyme obtained with pure IPTG (Supporting Information, Figure S1B). On the basis of these findings, the fermentation processes of the *E. coli BL21 (DE3) pLysS/pET28b(+)-hALOX15B/KANr* system and the *E.coli BL21 (DE3) pLysS/pET28b(+)-mAlox15/KANr* system were scaled up using 0.25 mM IPTG with 2.7% (by weight) lactose as an expression inducer, and the yield of ALOX expression was quantified using SDS-PAGE and Western blotting (Figure 1B, middle and lower panels). Under these conditions, 21.2% of hALOX15, 21.7% of hALOX15B, and 36.2% of mAlox15 were expressed in soluble form in the bacterial lysis supernatant (Supporting Information Figure S2).

In summary, our fermentation studies indicated that mammalian ALOX isoforms can be expressed in mg amounts per L liquid cultures (Table 1), and this data is consistent with a previous report on hALOX15 in the baculovirus/insect cell system [26]. In our comparative expression studies, hALOX15 was expressed in high amounts. The expression levels of hALOX15B and mAlox15 were substantially lower.

### 3.2. Purification of Recombinant Enzymes

**Figure 2:**
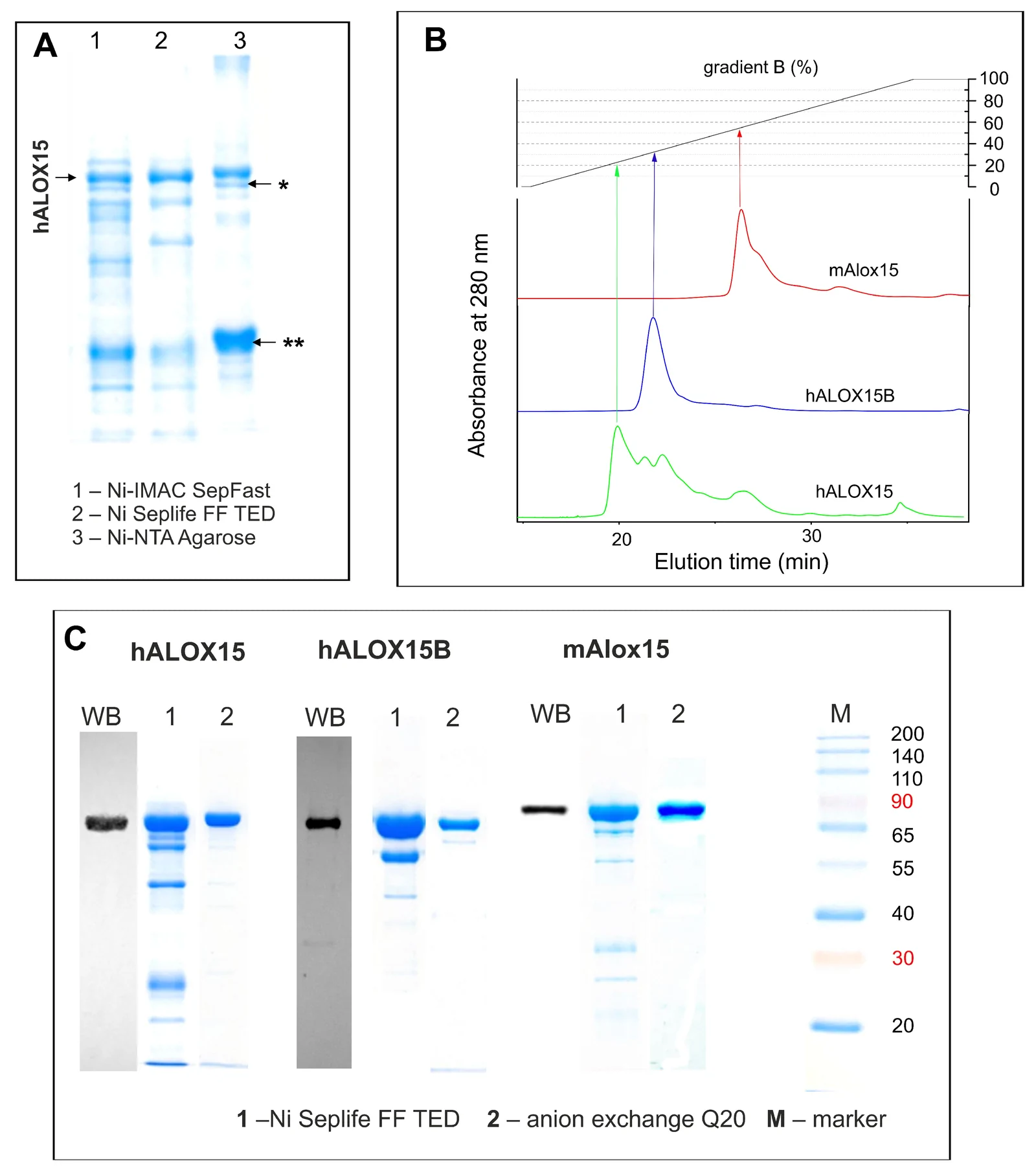
Chromatographic purification of recombinant ALOX15 proteins. (**A**) Comparative analysis of ALOX15 isolation and purification from cell supernatant using different affinity sorbents; *—bifunctional polymyxin resistance protein ArnA, **—unknown bacterial off-target protein. (**B**) Anion exchange chromatography of the purified ALOX species. FPLC was carried out on a BioPro SmartSep IEX Q20 (1 mL) column using a linear NaCl gradient. A280 of the column effluent and its conductivity were simultaneously recorded. (**C**) Comparative analysis of the purity of ALOX15 enzymes prepared by the two-step purification procedure on Ni Seplife FF TED and anion exchange chromatography on BioPro SmartSep IEX Q20. Protein purity analysis was performed by SDS-PAGE with Coomassie blue staining. Western blot analysis (WB) was carried out employing a horseradish peroxidase-conjugated monoclonal anti-his-tag antibody.

Ni-NTA is frequently used for purification of His-tag fusion proteins by affinity chromatography [30,31]. To optimize the purification procedure of recombinant hALOX15, we tested several commercial Ni-based resins. Here, we observed that co-incubation of bacterial lysates with Ni-NTA agarose or Ni-IMAC SepFast led to unspecific binding of non-His-tagged proteins. One of these non-target proteins was identified as bifunctional polymyxin resistance protein ArnA (Figure 2A, Supporting Information Figure S3), which was co-eluted with hALOX15 from the affinity matrix. Moreover, the use of Ni-IMAC SepFast as an affinity matrix was problematic, since we experienced a loss in catalytic activity. This inhibitory effect may be related to the architecture of the chromatographic matrix. The 3D structure of hALOX15 is flexible and the interaction of the N-terminal PLAT domain with the catalytic subunit may be important for protein stability. When the N-terminal His-tag interacts with the Ni-IMAC SepFast matrix, conformational alterations in the protein structure may be induced, and these structural changes might impact the catalytic activity. The best purification effect was observed when we employed Ni Seplife FF TED as an affinity matrix. Here, we detected a high degree of protein enrichment and the highest specific activity of the final enzyme preparation (Figure 2A).

Nevertheless, the protein fractions obtained from Ni Seplife FF TED affinity chromatography still contained minor amounts of off-target proteins (Figure 2C); thus, we decided to perform the second step of protein purification. For this purpose, the ALOX-positive affinity chromatography fractions were pooled, desalted, and then applied to a BioPro SmartSep IEX Q20 anion exchange column (Figure 2B). With this method, we reached a high (>98%) degree of electrophoretic homogeneity of the ALOX preparations. Interestingly, we observed that hALOX15B and mAlox15 required higher salt concentrations to be eluted from the matrix than hALOX15 (Figure 2B). This result suggests that hALOX15 carries a lower negative net charge at pH 8.0 than the other ALOX isoforms.

The elution profile of hALOX15B (Figure 2B) indicates a homogenous peak, and this data indicates that the enzyme is predominantly present as a single structural conformer. In contrast, the major elution peak of mAlox15 has a tail shoulder, and a small mAlox15 peak is eluted later on (32 min). Since SDS-PAGE of all fractions indicated the presence of 70 kDa proteins, it was concluded that the different retention behaviors in anion exchange chromatography may be related to a structural microheterogeneity of the mAlox15 protein. In other words, the protein occurs in different conformers, which can be separated from each other by anion exchange chromatography. This structural microheterogeneity was even more pronounced for hALOX15 (Figure S4). In addition to the major peak, which eluted with a retention time of about 20 min, we observed a number of additional ALOX15 peaks that were catalytically active. Thus, hALOX15 shows the highest degree of structural microheterogeneity.

Taken together, these data indicate that optimization of the cultivation parameters and purification procedures enabled the production of soluble forms of all three recombinant ALOX proteins under strictly comparable conditions.

### 3.3. Functional Characterization of Recombinant Enzymes

#### 3.3.1. Specific Catalytic Activity and Product Specificity

To prove the functionality of the recombinant ALOX isoforms, we first tested their catalytic activities with LA and AA as substrates, and the following kinetic constants were obtained for hALOX15: *k_cat_*^LA^ = 27.8 ± 1.3 s^−1^, K_M_^LA^ = 10.3 ± 1.0 µM, *k_cat_*^AA^ = 11.2 ± 0.8 s^−1^, K_M_^AA^ = 8.4 ± 1.6 µM for LA and AA, respectively. hALOX15B exhibited a lower K_M_ for AA than for LA (K_M_^LA^ = 19.8 ± 0.6 µM and K_M_^AA^ = 4.3 ± 2.1 µM), indicating a higher apparent affinity of the enzyme for AA. Consequently, its catalytic efficiency for AA oxygenation was approximately four-fold higher than that for LA, although the corresponding catalytic turnover rates were comparable (Table 2). The affinities of mAlox15 for the two fatty acids were higher than those of the human ortholog.

**Figure 3:**
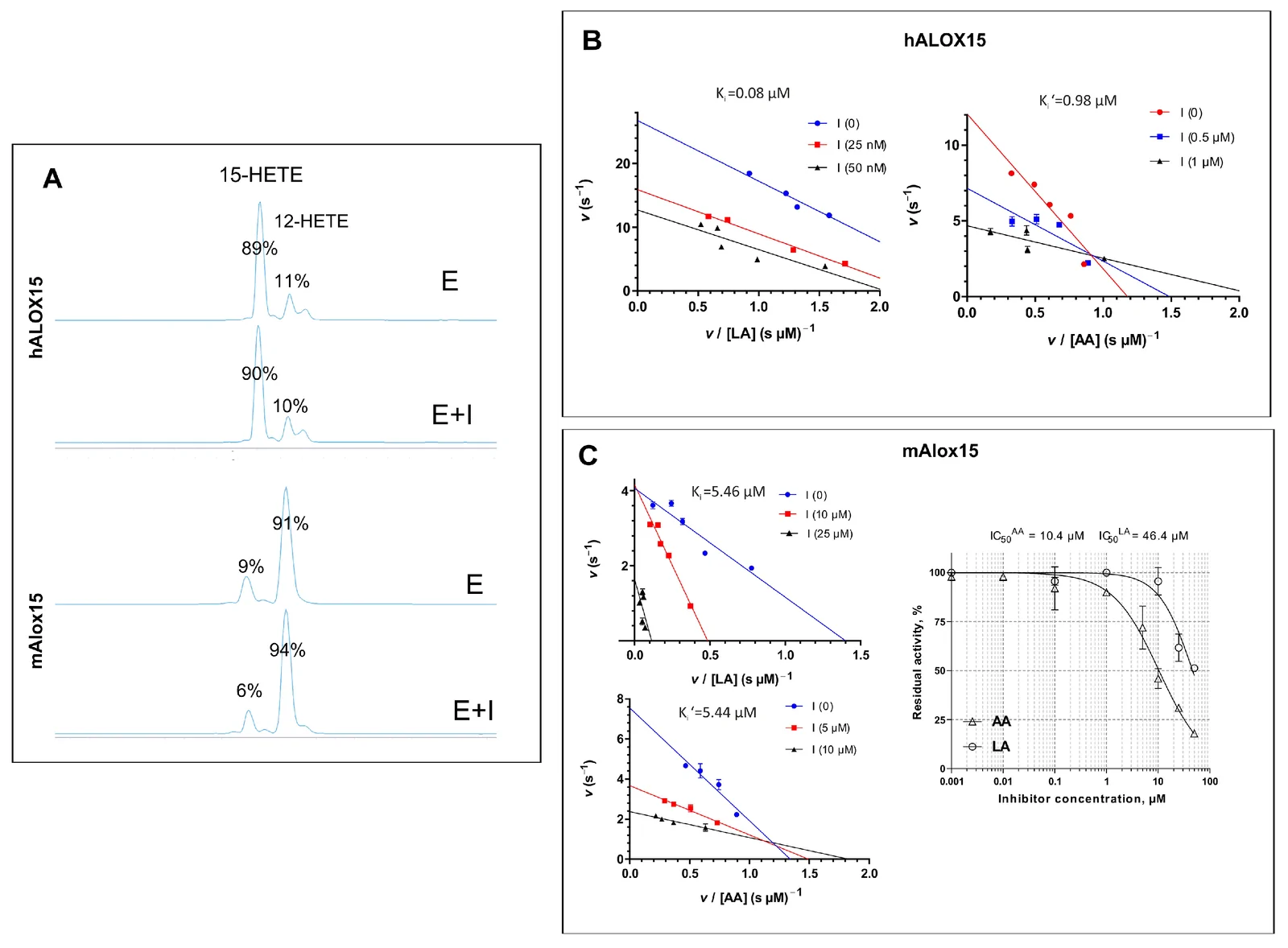
Product composition and mechanism of ALOX15 inhibition. (**A**) HPLC-MS analysis of the reaction products formed by recombinant human and mouse ALOX15 orthologs during incubation with AA as a substrate either in the presence (E+I) or absence (E) of the inhibitor. The inhibitor concentration applied was in the range of the K_i_ value of each enzyme. Oxygenation reactions were stopped by the addition of CH_3_CN (500 µL), and the reaction products were analyzed by RP-HPLC-MS, monitoring the ions with m/z 319 that correspond to the molecular ions of 12- and 15-HETE. The 15-HETE vs. 12-HETE ratios were normalized for the control incubations that did not contain enzyme. The minor peak that trails the 12-HETE peak represents 5R/S-HETE, which is present in small amounts as AA auto-oxidation product in our substrate solution. (**B**,**C**) Eadie–Hofstee plots demonstrating the effects of inhibitor on human (**B**) and mouse ALOX15 (**C**) when incubated at different concentrations of substrate fatty acids. Dose–response curves for inhibition of LA- and AA-oxygenase activities of mouse Alox15 at high substrate concentrations (50 µM) are shown ((**C**), (**right**) panel). Incubations were repeated at different inhibitor concentrations. All data points represent means ± SEM of three independent measurements (*n* = 3).

Pure human ALOX15 exhibits dual reaction specificity since AA is converted by this enzyme to a mixture of 15- and 12-H(p)ETE in a ratio of about 9:1 [34]. When we analyzed the product patterns of AA oxygenation catalyzed by our enzyme preparation, we obtained a 15-HpETE vs. 12-HpETE ratio of 89% vs. 11%. This data is consistent with previously reported results [35]. In contrast, our hALOX15B preparation oxygenated free AA almost exclusively to 15(S)–H(p)ETE, and this data confirms a previous report [33]. For the mouse Alox15 ortholog, a 15-HpETE vs. 12-HpETE ratio of 9 ± 1% vs. 91 ± 1% was calculated (Figure 3A).

#### 3.3.2. Enzyme Inhibition

To test the inhibitor sensitivity of our recombinant enzyme preparations, we used octyl (N-(5-(1H-indol-2-yl)-2-methoxyphenyl)sulfamoyl)carbamate as a mechanistic tool. This compound has previously been identified as a substrate-specific inhibitor of LA oxygenase activity by rabbit ALOX15, with hardly any effect on the AA oxygenase activity of this enzyme [27].

Similar to this previous data, we observed a pronounced substrate-selective inhibition of hALOX15 (Figure 3B). In fact, the LA oxygenase activity of this enzyme was strongly inhibited, but the K_M_ value remained unchanged (Supporting Information, Figure S5). However, for this enzyme, the mode of inhibition could not be described by a purely noncompetitive model, as was the case for rabbit ALOX15 [27]. In fact, the best fitting of the experimental data was obtained using a mixed inhibition model, and a K_i_ of 80.4 ± 4.12 nM was calculated (α = 0.64, R^2^ = 0.980) (Figure 3B). On the other hand, inhibition of the AA oxygenase activity of this enzyme was best described by an uncompetitive mode (decrease in both *k_cat_* and K_M_ values) with a K_i_^′^ of 0.98 ± 0.28 µM (R^2^ = 0.961) (Figure 3B, Supporting Information, Figure S5). Similar to hALOX15, the AA oxygenase activity of mAlox15 was strongly inhibited and both *k_cat_* and K_M_ values were strongly reduced (Supporting Information, Figure S6). The AA oxygenase activity of mAlox15 was best described by an uncompetitive inhibition model with a K_i_^′^ of 5.46 ± 0.36 µM (R^2^ = 0.992) (Figure 3C). On the other hand, inhibition of its LA oxygenase activity was best described by a mixed inhibition model (R^2^ = 0.854) with a K_i_ of 5.44 ± 1.41 µM (α = 4.8) (Figure 3C). Although these inhibition constants are close to each other, they were derived independently from separate datasets using different inhibition models. A marked increase in K_M_ was observed, whereas *k_cat_* remained largely unaffected (Supporting Information, Figure S6). This data suggests that the substrate and inhibitor compete for the same binding site at the enzyme. To support this hypothesis (Figure 3C, right panel), we analyzed the dose–response curves for inhibition of the LA- and AA-oxygenase activities of mAlox15 and quantified IC_50_ values (for LA, 10.4 µM; for AA, 46.4 µM). For a competitive mode of inhibition, the IC_50_ values strongly depend on the substrate concentration. We found that for AA oxygenation, there was no major difference between the K_i_ and IC_50_ values. In contrast, for LA, the IC_50_ was almost one order of magnitude higher than K_i_. Using the value obtained for LA, we calculated K_i_^′^ = 2.73 µM for LA on the basis of the Cheng–Prusoff equation. Both values appear to be close to each other, suggesting a competitive mode of inhibition.

#### 3.3.3. Anti-ALOX15/ALOX15B Antibodies Are Detected in the Blood of Patients Suffering from Colorectal Cancer

**Figure 4:**
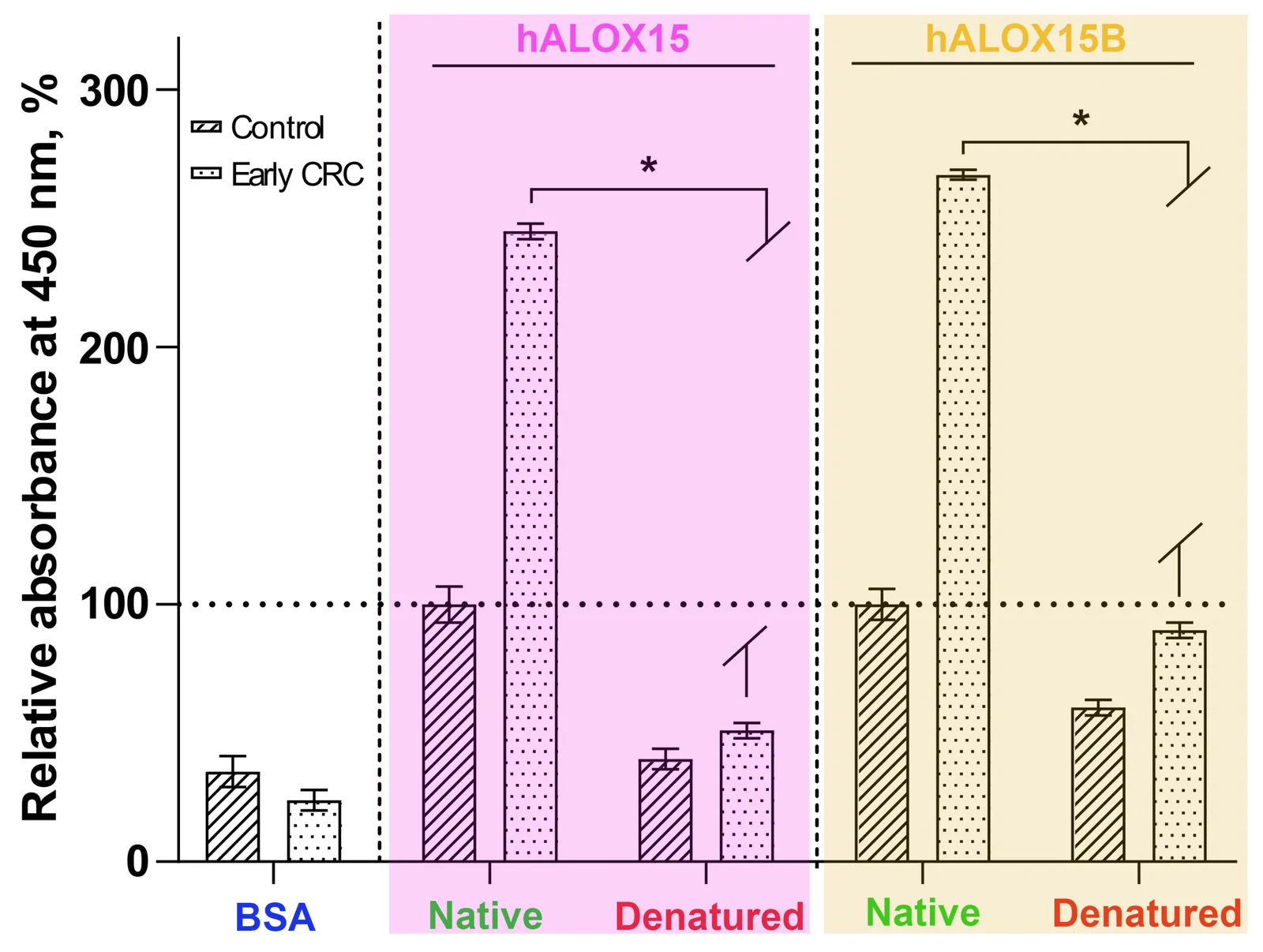
Occurrence of antibodies cross-reacting with hALOX15 and hALOX15B in the blood serum of patients suffering from colorectal cancer. Quantitative ELISAs were performed as described in Materials and Methods. Denatured hALOX15 and hALOX15B were prepared by gradually heating the enzyme solutions to 80 °C for 30 min. The small amount of precipitate was spun down, and the concentration of remaining protein was quantified spectrophotometrically. Bovine serum albumin (BSA) was used as a negative control. The controls represent the mean value ± SEM of six control sera from non-cancer patients. The serum samples also represent the mean value ± SEM obtained from six patients in early stages (I and II) of colorectal cancer (CRC). Since the same serum samples were tested against native and denatured ALOX isoforms, they are considered paired observations. Owing to the small sample size (*n* = 6), we employed the two-sided Wilcoxon matched-pairs signed-rank test (hALOX15: W = −21, *p* = 0.0313; hALOX15B: W = −21, *p* = 0.0313) for statistical evaluation of the experimental raw data. Visualization of the data was carried out by GraphPad Prism (* *p* < 0.05).

Since ALOX15 and ALOX15B have been implicated in cancer progression, we tested serum samples from healthy volunteers and patients suffering from colorectal cancer for the presence of antibodies that cross-react with recombinant human ALOX15 (hALOX15) and recombinant human ALOX15B (hALOX15B), and the data are shown in Figure 4. For these experiments, bovine serum albumin (BSA) was used as a negative control antigen. Here, we found that the blood serum of cancer patients involved antibodies that cross-reacted with recombinant hALOX15 and hALOX15B. This cross-reactivity was strongly reduced with the control antigen. Most interestingly, this difference was not observed when the ALOX isoforms were heat-denatured. In these experiments, we did not see any immunological cross-reactivity. These findings suggested that antibodies directed against human ALOX15 and ALOX15B may be present in the blood serum of patients suffering from early forms of colorectal cancer; thus, quantification of the anti-ALOX15/ALOX15B titers in human individuals may be used as a diagnostic method to screen for patients suffering from the early stages of colorectal cancer, which do not display other symptoms of the disease such as intestinal bleeding. However, for the time being, it remains unclear whether the antibodies are produced against the two ALOX isoforms or whether they are produced against unknown proteins but cross-react with the two ALOX isoforms. Moreover, it remains unclear whether the anti-ALOX15 antibodies in the blood serum of cancer patients cross-react with ALOX15B or whether separate antibodies directed against ALOX15 and ALOX15B are present. Finally, our enzyme preparations can also be used to test whether patients suffering from other types of cancer, such as prostate and ovarian cancer, may also carry antibodies cross-reacting with hALOX15 and ALOX15B. These clinically relevant questions may be addressed in more detailed follow-up studies.

## 4. Discussion

The patho-physiological functions of ALOX isoforms in human diseases have made these enzymes promising targets for pharmacological research. In recent years, a large number of ALOX inhibitors have been discovered [36,37]. However, in many of these studies, neither the isoform specificity (inhibitor potency against human ALOX15 vs. human ALOX15B) nor the ortholog specificity (inhibitor potency against hALOX15 vs. mAlox15) has been tested. Moreover, in many studies, the inhibitory potency of a given compound for pure enzymes has been compared with that of crude enzyme preparations, and such “unfair” comparisons may frequently lead to misinterpretations of the inhibitor specificity. Moreover, the functional differences between hALOX15 and mAlox15 have frequently not been considered.

The formation of inclusion bodies, which mainly consist of misfolded recombinant proteins, is a major complication of the bacterial expression of recombinant proteins. In this study, we observed the formation of catalytically inactive and insoluble proteins, and this process was dominant during the early phases of the expression period (Supporting Information, Figure S2). Despite numerous attempts to reduce the share of inclusion body formation, we could not prevent this process. For instance, we created a hybrid ALOX15 expression plasmid, which involved a secretion sequence [38] in addition to the hALOX15 coding sequence. Although the fusion protein was nicely expressed, it was only present in the bacterial lysate pellet and lacked any ALOX activity. Instead, fermentation studies indicated that soluble forms of mammalian ALOX isoforms can be best expressed using an IPTG–lactose mixture in mg amounts per L liquid cultures (Table 1).

Ni-NTA resins are frequently used for purification of His-tag fusion proteins by affinity chromatography [30,31]. However, the purification of recombinant ALOX15 may also be challenging due to its structural flexibility [11]. When we tested several commercial Ni-based resins, we found that Ni-IMAC SepFast not only nonspecifically binds non-target proteins, but also inactivates hALOX15 eluted from the affinity matrix. This effect may be related to the architecture of the chromatographic matrix and the resulting protein–matrix interactions. hALOX15 is a conformationally flexible enzyme, and the interaction of its N-terminal PLAT domain and catalytic domain may be important for protein stability [11]. Sorption of the N-terminal His-tag on the mechanically robust and porous Ni-IMAC SepFast matrix, together with additional nonspecific contacts between the enzyme and the resin, may induce conformational changes and result in the formation of unproductive enzyme conformations. However, additional studies are required to investigate matrix-induced conformational changes in hALOX15.

During functional characterization of recombinant enzymes (hALOX15, hALOX15B and mAlox15) obtained under similar conditions, we compared their catalytic efficiencies. The following conclusions could be drawn: (i) Human ALOX15 prefers LA as an oxygenation substrate over AA. In fact, the *k_cat_*/K_M_ ratio for LA was more than 2-fold higher than the corresponding ratio for AA. (ii) hALOX15B strongly prefers AA over LA. (iii) For mouse Alox15, the catalytic efficiencies for both PUFAs were similar. This data indicates that when compared with mAlox15, the human ortholog exhibits improved catalytic activity with LA, and this kinetic difference may be considered as a driving force for the evolutionary shift in the reaction specificity from AA 12-lipoxygenating to AA 15-lipoxygenating enzymes during late mammalian evolution [6,39]. These data are further supported by our inhibitor studies. In fact, octyl (N-(5-(1H-indol-2-yl)-2-methoxyphenyl)sulfamoyl)carbamate effectively inhibits the AA-oxygenase activity of both human and mouse ALOX15 orthologs in the low micromolar range. Kinetic analysis indicates strong inhibitor binding to the enzyme–substrate complex (ES^AA^), promoting the formation of a non-productive ternary complex (ESI^AA^) for both ALOX15 orthologs. Within a small error range, the inhibitor did not modify the product pattern of AA oxygenation by the two enzymes (Figure 3A). In contrast, the mechanism of inhibition of the LA oxygenase activities of the two enzymes was different (Figure 3B,C). Here, we observed a mixed type of inhibition for hALOX15 but a competitive type of inhibition for mAlox15. The potency of the inhibitor was almost two orders of magnitude higher for the LA-oxygenase reaction of hALOX15 (K_i_ = 80.4 nM) when compared to mAlox15 (K_i_ = 5.4 µM).

## 5. Conclusions

The proposed methodology allows the preparation of functional recombinant ALOX isoforms in mg amounts. The recombinant proteins are suitable for high-throughput inhibitor screens, for kinetic measurements, and also for structural studies exploring enzyme–ligand interactions. Owing to the functional differences between mouse and human ALOX15 orthologs (hALOX15 is an AA 15-lipoxygenating enzyme, mAlox15 is AA 12-lipoxygenating), the ortholog specificity of newly developed ALOX15 inhibitors should always be tested, and the presented expression protocol allows the preparation of sufficient amounts of mouse and human ALOX15.

## Figures and Tables

**Table 1:** Expression levels of ALOX isoforms in E. coli BL21 (DE3) pLysS cells with IPTG–lactose as inducer in a 7 L laboratory fermenter.

Protein	OD^600^	Amounts of Wet Biomass (g/L)	Amounts of Soluble Protein Expressed per 1 L Liquid Culture (mg/L) *
hALOX15	13.0	14.2	100
hALOX15B	27.5	28.5	34
mAlox15	7.3	16.8	15

* ALOX protein amounts were evaluated by quantitative Western blotting using aliquots of purified hALOX15 as a reference compound.

**Table 2:** Kinetic parameters of ALOX15 enzymes. hALOX15 (2 μg pure protein), hALOX15B (4 μg pure protein), mAlox15 (2.6 μg pure protein) were incubated in PBS with different concentrations of AA or LA, and oxygenation kinetics were assayed spectrophotometrically (reaction volume 1 mL). The reaction was started by addition of substrate. No detergents were used. The kinetic constants (K_M_ and *k_cat_*) were evaluated, and the *k_cat_*/K_M_ ratios were calculated as a suitable measure for the catalytic efficiency. All data represent mean values ± SD of three independent measurements (*n* = 3).

Enzyme		LA			AA	
	*k_cat_* (s^−1^)	K_M_ (μM^−^)	*k_cat_*/K_M_ (s^−1^ μM^−1^)	*k_cat_* (s^−1^)	K_M_ (μM)	*k_cat_*/K_M_ (s^−1^ μM^−1^)
hALOX15	27.8 ± 1.3	10.3 ± 1.0	2.70 (3.79) [24]	11.2 ± 0.8	8.4 ± 1.6	1.33 (1.44) * [32]
hALOX15B	0.9 ± 0.1	19.8 ± 0.6	0.05	0.9 ± 0.2	4.3 ± 2.1	0.21 (0.26) [33]
mAlox15	4.1 ± 0.2	3.1 ± 0.7	1.32	7.2 ± 0.7	5.1 ± 1.2	1.41

* previously obtained values are in the parentheses and corresponding references are given.

## Data Availability

The original experimental raw data presented in this study can be obtained upon request from I.I.

## Supplementary Materials

The following supporting information can be downloaded at: https://www.mdpi.com/article/10.3390/metabo16080599/s1, Figure S1: Bacterial expression of hALOX15; Figure S2: Expression of recombinant hALOX15 proteins; Figure S3: Primary amino acid sequence analysis of bacterial off target protein cleavage peptides; Figure S4: Purification of human ALOX15 by anion exchange chromatography; Figure S5: Kinetic parameters of human ALOX15; Figure S6: Kinetic parameters of mouse Alox15.

## Abbreviations

The following abbreviations are used in this manuscript:

ALOXs	arachidonic acid lipoxygenases
PUFAs	polyunsaturated fatty acids
AA	arachidonic acid
LA	linoleic acid
PPARγ	peroxisome proliferator-activated receptor γ
PLAT domain	Polycystin-1, Lipoxygenase, Alpha-Toxin domain
13(S)-H(p)ODE	13(S)-hydro(pero)xy-9Z,11E-octadecadienoic acid
15(S)-H(p)ETE	15(S)-hydro(pero)xy-5Z,8Z,11Z,13E-eicosatetraenoic acid
12(S)-H(p)ETE	12(S)-hydro(pero)xy-5Z,8Z,10E,14Z-eicosatetraenoic acid
SDS-PAGE	sodium dodecylsulfate polyacrylamide gel electrophoresis
ELISA	enzyme-linked immunosorbent assay
EDTA	ethylenediaminetetraacetic acid
BSA	bovine serum albumin
IPTG	isopropyl β-d-1-thiogalactopyranoside
IC_50_	half-maximal inhibitory concentration
CRC	colorectal cancer
DMSO	dimethyl sulfoxide
PBS	phosphate-buffered saline
WB	Western blot analysis
FPLC	Fast Protein Liquid Chromatography
HPLC	High-Performance Liquid Chromatography
OD^600^	optical density at 600 nm
