# Supplementary material for: Mammalian Arachidonic Acid 15-Lipoxygenases: Fed-Batch Fermentation, Enzyme Purification and Functional Characterization
Source: Metabolites. 2026 Aug 21;16(8):599. doi: 10.3390/metabo16080599 (PMC13515856; doi:10.3390/metabo16080599)
Supplement: Supplementary file 1 [file metabolites-16-00599-s001.zip › metabolites-4440018-supplementary.pdf]

1. Bacterial expression of human ALOX15.

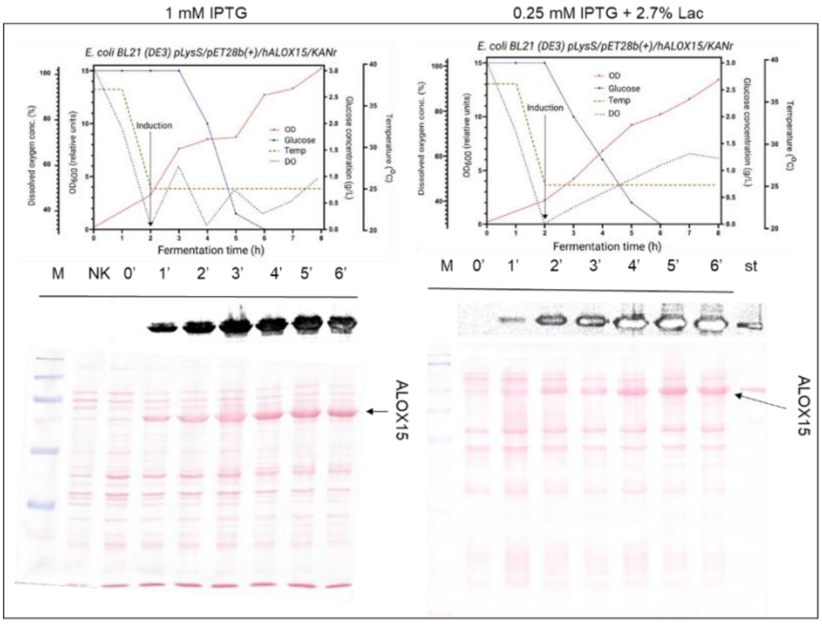

A

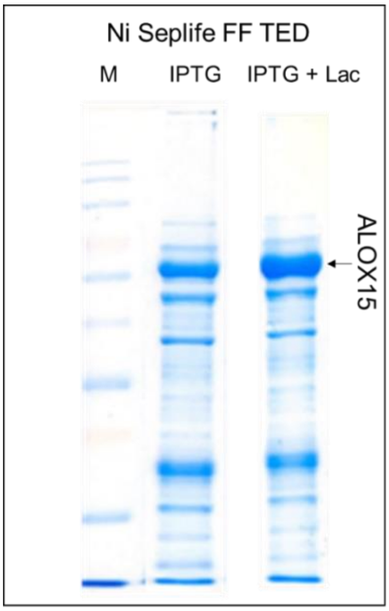

B

Figure S1. Bacterial expression of hALOX15. (A) Bacterial expression of hALOX15 as N-terminal his-tag fusion protein with IPTG (left upper panel) and a mixture of IPTG + lactose (right upper panel) as inducers in a 7 L laboratory fermenter. Aliquots of the fermentation sample were taken at different time points and SDS-PAGE was carried out. Protein blots were reversible stained with Ponceau red (lower panel) and for Western-blotting a horseradish peroxidase-conjugated monoclonal antibody against the his-tag was used (middle panel). Legend: st – pure recombinant hALOX15 enzyme as a standard; 0'–6' – induction time. (B) Comparative analysis of the two ALOX15 preparations purified by Ni Seplife FF TED. Application of IPTG-lactose mixture resulted in an increase of enzyme yield when compared to pure IPTG.

2. Time course of bacterial expression of different ALOX isoforms

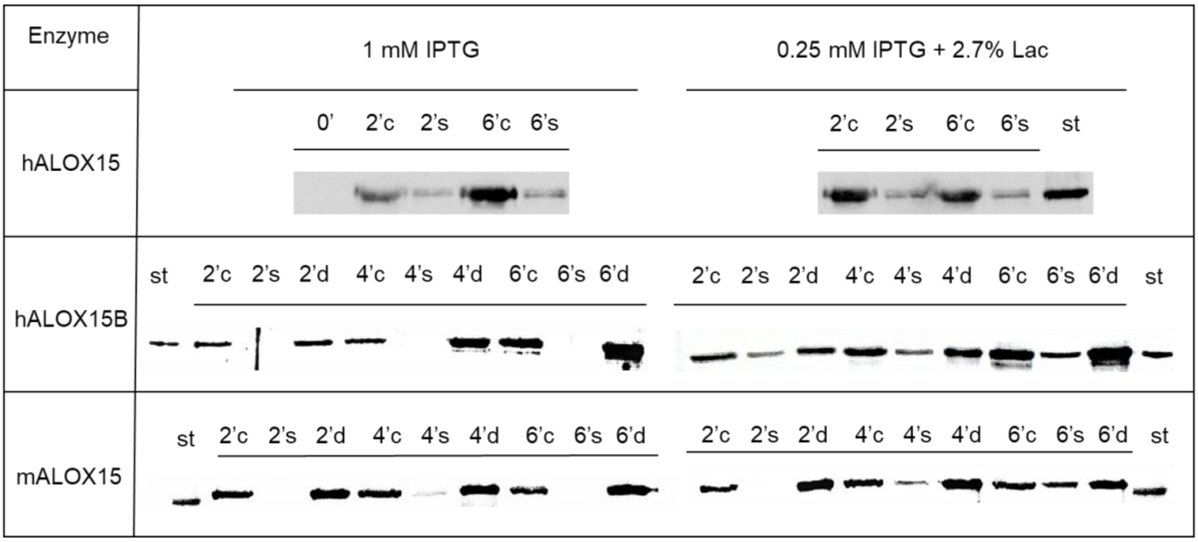

Figure S2. Expression of recombinant hALOX15 proteins. Comparative expression of recombinant ALOX isoforms. ALOX isoforms were expressed as N-terminal his-tag fusion protein and IPTG (left panel) or a mixture of IPTG + lactose (right panel) were used as expression inducers. Aliquots of the fermentation sample were taken at different time points and the presence of ALOX proteins in the bacterial lysis supernatant and the bacterial lysis pellet were quantified by Western blotting: 0'–6' – induction time in hours, c – total cell suspension, s – bacterial lysate supernatant, d – debris (bacterial lysate pellet). As shown by immunoblotting inactive and insoluble aggregates of the ALOX15 isoforms are formed within the first hours of induction.

3. Protein-chemical analysis of bacterial bifunctional polymyxin resistance protein ArnA

ARNA_ECOBW **Mass:** 74242 **Score:** 349 **Expect:** 2.9e-31 **Matches:** 42

Bifunctional polymyxin resistance protein ArnA OS=Escherichia coli (strain K12 / MC4100 / BW2952) OX=595496 GN=arnA PE=3 SV=1

Observed	Mr(expt)	Mr(calc)	ppm	Start - End	Miss	Peptide
754.3549	753.3476	753.3480	-0.44	536 - 541	0	R.CFTDIR.D
825.3992	824.3919	824.3851	8.34	536 - 541	0	R.CFTDIR.D + Propionamide (C)
870.4567	869.4494	869.4395	11.3	40 - 47	0	K.AFYGSVAR.L
886.4808	885.4735	885.4668	7.61	550 - 557	0	R.IIENAGNR.C
936.4843	935.4770	935.4712	6.19	542 - 549	0	R.DGIEALYR.I
961.4539	960.4467	960.4375	9.56	629 - 635	0	R.CLDWEPK.I + Propionamide (C)
1003.4693	1002.4620	1002.4519	10.1	612 - 619	0	K.GYQDVEHR.K
1004.4834	1003.4762	1003.4698	6.34	490 - 497	0	R.PFNWMGPR.L
1020.4817	1019.4744	1019.4647	9.48	490 - 497	0	R.PFNWMGPR.L + Oxidation (M)
1084.6140	1083.6068	1083.6036	2.87	139 - 149	0	R.ADAGAIVAQLR.I
1097.4834	1096.4761	1096.4607	14.0	192 - 200	0	R.ENEATCFGR.R + Propionamide (C)
1110.6019	1109.5947	1109.5869	6.95	481 - 489	0	K.EGLQFTLFR.P
1118.5431	1117.5358	1117.5291	5.96	602 - 611	0	R.VVESSSYYGK.G
1131.5653	1130.5581	1130.5502	6.93	304 - 313	0	R.LNSQPACTAR.R + Propionamide (C)
1150.6418	1149.6345	1149.6254	7.91	182 - 191	0	K.HGNILEIAQR.E
1212.6151	1211.6079	1211.5989	7.42	592 - 601	0	R.HHFPPFAGFR.V
1240.7203	1239.7131	1239.7047	6.71	138 - 149	1	K.RADAGAIVAQLR.I
1253.7568	1252.7495	1252.7391	8.35	171 - 181	0	R.QLLEQTLPAIK.H
1410.6950	1409.6877	1409.6827	3.55	405 - 415	0	R.VFELDFEENLR.I
1530.7387	1529.7314	1529.7263	3.37	201 - 212	1	R.RTPDDSFLEWHK.P
1627.9167	1626.9095	1626.9093	0.09	150 - 164	0	R.IAIAPDDIAITLHHK.L
1709.0142	1708.0070	1708.0134	-3.80	511 - 526	0	R.AITQLILNLVEGSPIK.L
1742.8452	1741.8380	1741.8457	-4.46	536 - 549	1	R.CFTDIRDGIEALYR.I + Propionamide (C)
1806.8737	1805.8664	1805.8737	-4.02	223 - 239	0	R.AVADPWPGAFSYVGNQK.F
1818.9309	1817.9236	1817.9352	-6.38	71 - 85	0	R.IAQLSPDVIFSFYYR.H
1847.8479	1846.8407	1846.8481	-4.04	427 - 442	0	R.IIFPSTSEVYGMCSDK.Y + Propionamide (C)
1852.0301	1851.0228	1851.0367	-7.50	318 - 334	0	R.VLILGVNGFIGNHLTER.L
1985.9340	1984.9267	1984.9452	-9.30	636 - 651	0	K.IDMQETIDETLDFFLR.T
2038.9450	2037.9377	2037.9279	4.81	338 - 355	0	R.EDHYEVYGLDIGSDAISR.F
2044.0910	2043.0837	2043.1074	-11.60	383 - 400	0	K.CDVVLPLVAIATPIEYTR.N + Propionamide (C)
2076.0510	2075.0437	2075.0589	-7.29	53 - 70	0	R.GIPVYAPDNVNHPLWVER.I
2096.0228	2095.0155	2095.0462	-14.66	481 - 497	1	K.EGLQFTLFRPFNWMGPR.L
2100.0538	2099.0465	2099.0436	1.40	443 - 460	1	K.YFDEDHSNLIVGPVNKPR.W
2206.1411	2205.1338	2205.1542	-9.25	115 - 134	0	R.APLNWVLVNGETETGVTLHR.M
2421.2208	2420.2135	2420.1972	6.76	335 - 355	1	R.LLREDHYEVYGLDIGSDAISR.F
2479.2265	2478.2193	2478.2114	3.17	202 - 222	1	R.TPDDSFLEWHKPASVLHNMVR.A
2635.3335	2634.3262	2634.3125	5.20	201 - 222	2	R.RTPDDSFLEWHKPASVLHNMVR.A
2730.5076	2729.5003	2729.4904	3.62	86 - 110	0	R.HLIYDEILQLAPAGAFNLHGSLLPK.Y
2928.4821	2927.4748	2927.3721	35.1	629 - 651	1	R.CLDWEPKIDMQETIDETLDFFLR.T + Propionamide (C)
3217.6016	3216.5943	3216.5570	11.6	356 - 381	0	R.FLNHPHFHFVEGDISIHSEWIEYHVK.K
3335.6252	3334.6179	3334.5751	12.8	625 - 651	2	R.NAHRCLDWEPKIDMQETIDETLDFFLR.T
4919.7196	4918.7123	4918.5533	32.3	254 - 303	0	K.AQPGSVISVAPLLIACGDGALEIVTGQAGDGITMQGSQLAQTLGLVQGSR.L

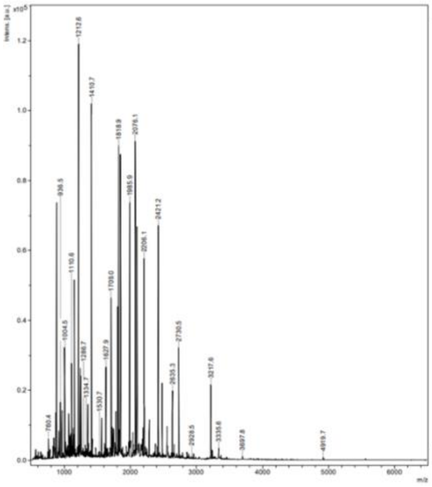

Protein sequence coverage: 72%

Matched peptides shown in ***bold red***.

```
  1 MKTVVFAYHD MGCLGIEALL AAGYEISAIF THTDNPGEKA FYGSVARLAA
 51 ERGIPVYAPD NVNHPLWVER IAQLSPDVIF SFYYRHLIYD EILQLAPAGA
101 FNLHGSLLPK YRGRAPLNWV LVNGETETGV TLHRMVKRAD AGAIVAQLRI
151 AIAPDDIAIT LHHKLCHAAR QLLEQTLPAI KHGNILEIAQ RENEATCFGR
201 RTPDDSFLEW HKPASVLHNM VRAVADPWPG AFSYVGNQKF TVWSSRVHPH
251 ASKAQPGSVI SVAPLLIACG DGALEIVTGQ AGDGITMQGS QLAQTLGLVQ
301 GSRLNSQPAC TARRRTRVLI LGVNGFIGNH LTERLLREDH YEVYGLDIGS
351 DAISRFLNHP HFHFVEGDIS IHSEWIEYHV KKCDVVLPLV AIATPIEYTR
401 NPLRVFELDF EENLRIIRYC VKYRKRIIFP STSEVYGMCS DKYFDEDHSN
451 LIVGPVNKPR WIYSVSKQLL DRVIWAYGEK EGLQFTLFRP FNWMGPRLDN
501 LNAARIGSSR AITQLILNLV EGSPIKLIDG GKQKRCFTDI RDGIEALYRI
551 IENAGNRCDG EIINIGNPEN EASIEELGEM LLASFEKHPL RHHFPPFAGF
601 RVVESSSYYG KGYQDVEHRK PSIRNAHRCL DWEPKIDMQE TIDETLDFFL
651 RTVDLTDKPS
```

Figure S3. Primary amino acid sequence analysis of bacterial off target protein cleavage peptides. Amino acid sequence analysis of bacterial bifunctional polymyxin resistance protein ArnA cleavage peptides obtained by MALDI-TOF-MS. Red color indicates peptides that found match in the primary sequence protein

4. Conformational heterogeneity of hALOX15.

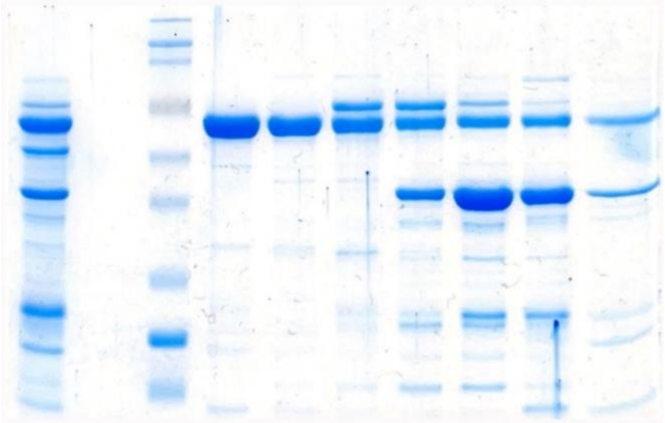

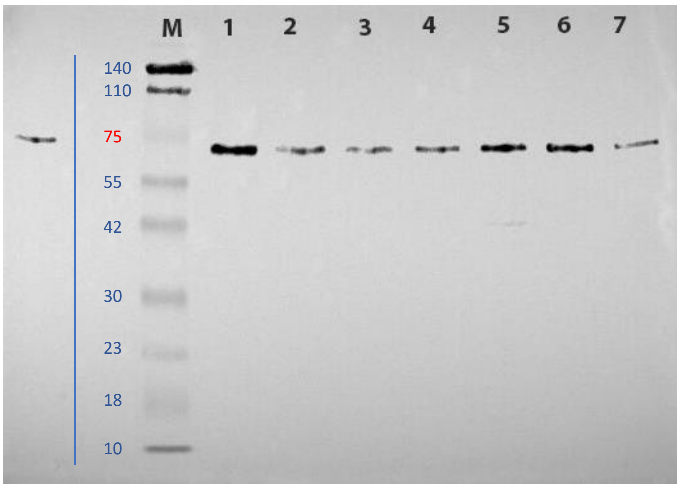

Figure S4. Purification of human ALOX15 by anion exchange chromatography on BioPro SmartSep IEX Q20 (see Fig. 2B, lower panel). The elution fractions between 19.5 and 23.5 min were collected and analyzed by either SDS electrophoresis followed by Coomassie blue staining or by immunoblotting with horseradish peroxidase-conjugated rabbit monoclonal antibodies against the his-tag.

5. Kinetic measurements of human and mouse ALOX15 orthologs with and without inhibitor.

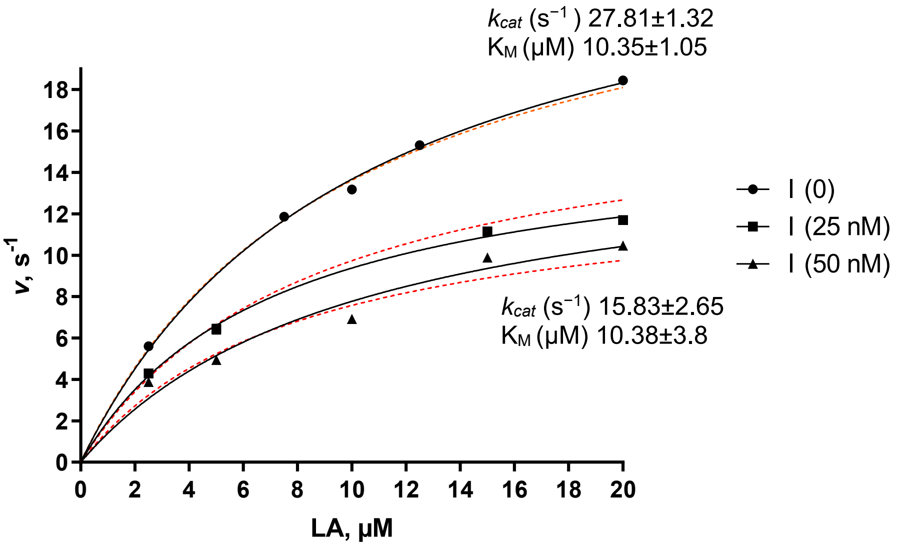

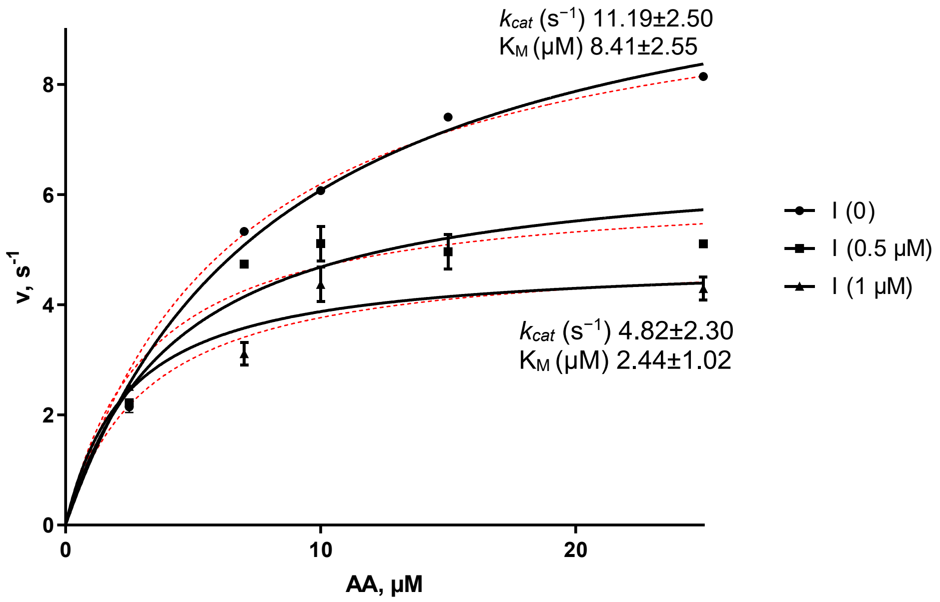

Figure S5. Kinetic parameters of human ALOX15.

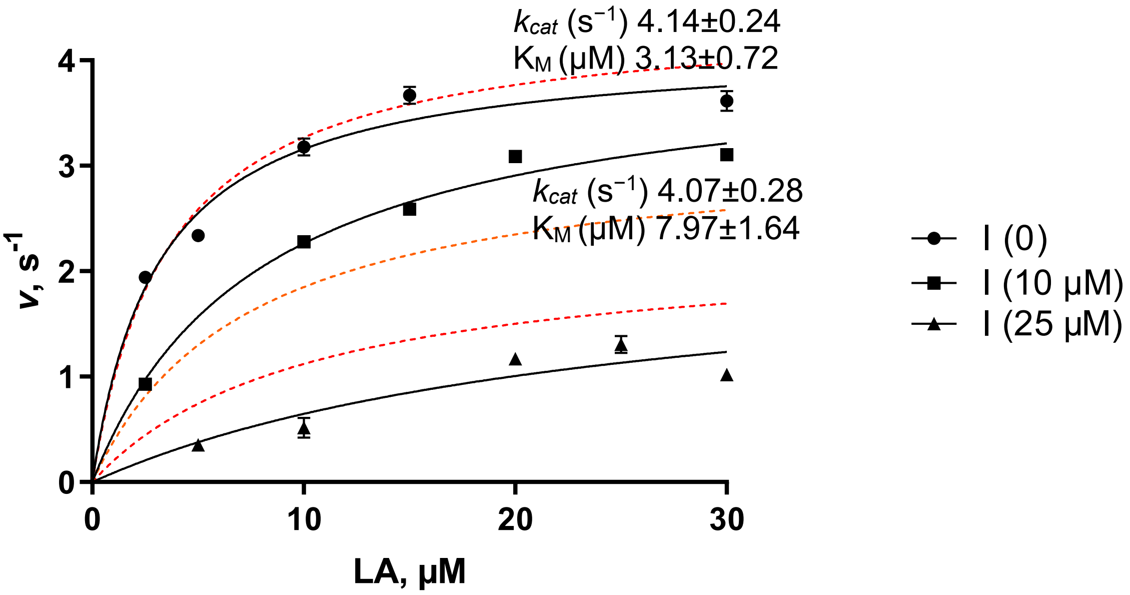

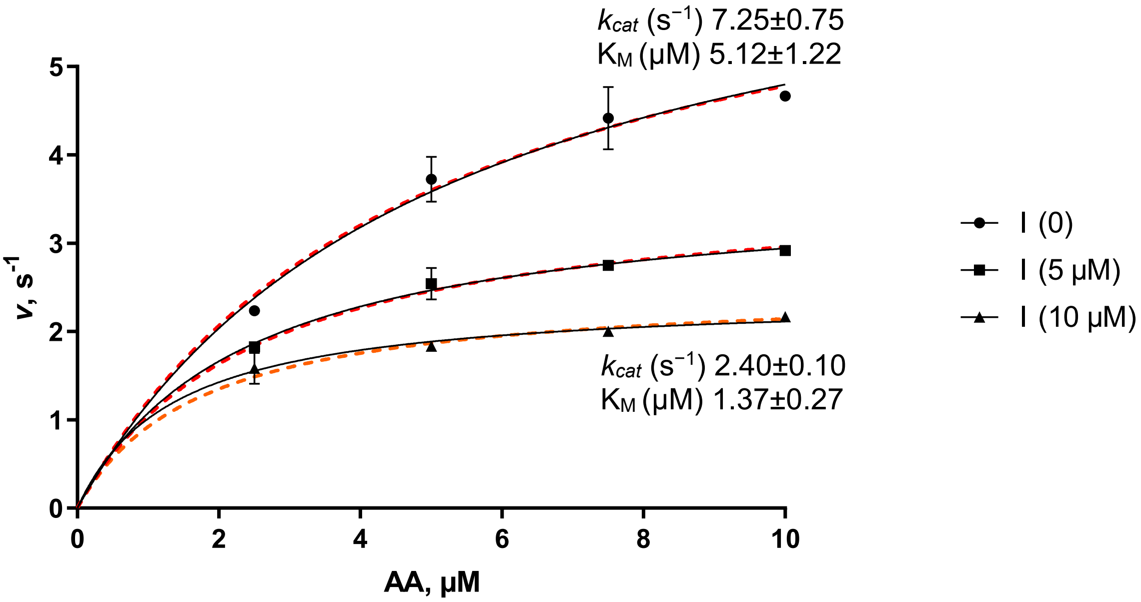

Figure S6. Kinetic parameters of mouse Alox15.
